# Hollow Carbon Sphere Nanoreactors Loaded with PdCu Nanoparticles: Void‐Confinement Effects in Liquid‐Phase Hydrogenations

**DOI:** 10.1002/anie.202007297

**Published:** 2020-08-18

**Authors:** Chao Dong, Qun Yu, Run‐Ping Ye, Panpan Su, Jian Liu, Guang‐Hui Wang

**Affiliations:** ^1^ Key Laboratory of Biofuels Qingdao Institute of Bioenergy and Bioprocess Technology Chinese Academy of Sciences Qingdao 266101 China; ^2^ State Key Laboratory of Catalysis Dalian Institute of Chemical Physics Chinese Academy of Sciences Dalian 116023 China; ^3^ University of Chinese Academy of Sciences Beijing 100049 China; ^4^ Dalian National Laboratory for Clean Energy Dalian 116023 China; ^5^ DICP-Surrey Joint Centre for Future Materials Department of Chemical and Process Engineering, and Advanced Technology Institute University of Surrey Guilford GU2 7XH UK

**Keywords:** heterogeneous catalysis, hollow nanoreactors, hydrogenation, shape-selective catalysis, void-confinement effects

## Abstract

Nanoreactors with hollow structures have attracted great interest in catalysis research due to their void‐confinement effects. However, the challenge in unambiguously unraveling these confinement effects is to decouple them from other factors affecting catalysis. Here, we synthesize a pair of hollow carbon sphere (HCS) nanoreactors with presynthesized PdCu nanoparticles encapsulated inside of HCS (PdCu@HCS) and supported outside of HCS (PdCu/HCS), respectively, while keeping other structural features the same. Based on the two comparative nanoreactors, void‐confinement effects in liquid‐phase hydrogenation are investigated in a two‐chamber reactor. It is found that hydrogenations over PdCu@HCS are shape‐selective catalysis, can be accelerated (accumulation of reactants), decelerated (mass transfer limitation), and even inhibited (molecular‐sieving effect); conversion of the intermediate in the void space can be further promoted. Using this principle, a specific imine is selectively produced. This work provides a proof of concept for fundamental catalytic action of the hollow nanoreactors.

## Graphical abstract

**A pair of hollow carbon sphere** (HCS) nanoreactors with PdCu nanoparticles encapsulated inside the HCS (PdCu@HCS) and supported outside the HCS (PdCu/HCS) have been prepared. Based on the two comparable systems, the void‐confinement effects of hollow nanoreactors in liquid‐phase hydrogenation have been extensively investigated in a two‐chamber reactor.

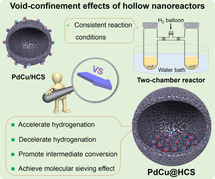

Hollow structured nanomaterials containing a void space inside a distinct shell have attracted broad interest in the fields of catalysis, energy storage and conversion, biomedicine, etc.,^1^ due to the specific properties determined by their unique structures (e.g., high surface area, accessible void space, tunable porous structure of the shell). Especially in catalysis, hollow structured nanomaterials in which the active species are loaded in different spatial regions (e.g., exterior/interior surface of the shell, in the shell, in the void space) can be considered as nanoreactors from the perspective of chemical and biomolecular engineering, which has been utilized in a series of heterogeneous catalytic processes.^2^ It is believed that the shell of these hollow nanoreactors, which often display an enriched porous structure and high surface area, can sustain a high loading of the active species.^3^ By encapsulating the active species inside the shell, the hollow nanoreactors can protect them against sintering, leaching, and aggregating even under harsh reaction conditions.^4^ In addition, the hollow nanoreactors can provide molecular‐sieving capability to realize size‐selective and poison‐resistant catalysis.^5^ Moreover, the void space of the hollow nanoreactors can provide a unique nanospace for storage or confinement of the reactant molecules, which probably induces an accumulation of the reactant molecules and locally alters the hydrodynamics of the nanoreactors (e.g., increasing the retention time of reactant molecules in the cavity).^5^ Besides these advantages, the hollow nanoreactors can be used as ideal models for investigating catalytic mechanisms at nanoscale and guiding the rational design of efficient catalysts for practical applications.

During the past decades, many studies have been devoted to unraveling the catalytic properties, in particular the void‐confinement effects, of the hollow nanoreactors in liquid‐phase reactions.^2^, ^3^, ^4^, ^5^ In many cases, enhanced catalytic activities or tunable selectivity in reactions over the hollow nanoreactors have been observed compared with their nonhollow analogues, which are ascribed to the void‐confinement effects or molecular‐sieving effects induced by the hollow structures.^3^, ^4^, ^5^ The excellent catalytic performances are also often attributed to the void‐confinement effects without any comparative analysis, solely because of the existence of the void spaces. In fact, it is not easy to clearly address the void‐confinement effects even after comparative analysis with the nonhollow materials, because many other factors are also highly influential for the catalytic properties. For instance, compared with the nonhollow analogues, the synthesis procedures to fabricate the hollow structured nanoreactors can potentially introduce other modifications in the shell structures as well as the compositions/morphologies of the active species which might further contribute to differences in the catalytic performance.^5^ In addition, it remains a challenge to precisely control the consistency of the reaction conditions (e.g., pressure, temperature, stirring speed) during the individual catalytic tests of the hollow nanoreactors and their nonhollow analogues, which may introduce factors that can also affect the catalytic performance. Therefore, providing both the comparative nanomaterials and reaction system with consistent reaction conditions to decouple the void‐confinement effects from other factors is highly required to unambiguously ascribe and understand the void‐confinement effects of the hollow structured nanoreactors.

Herein, we report a general strategy to synthesize a pair of hollow nanoreactors with the presynthesized PdCu bimetallic nanoparticles encapsulated in the HCS (PdCu@HCS) and supported outside of the HCS (PdCu/HCS), respectively, while the other structural features are the same for both systems. Based on these two comparative nanoreactors, it is quite possible to deeply study the void‐confinement effects of the hollow nanoreactors in catalysis. The strategy for preparing the pair of nanoreactors is illustrated in Figure 1. Firstly, the PdCu nanoparticles with a diameter of 2.8±0.2 nm (Figure S1a,b) are synthesized by a solvothermal method reported previously with a minor modification.^6^ Next, for the synthesis of PdCu@HCS (path A), the PdCu nanoparticles are introduced into P123/oleic acid emulsion droplets, by mixing a P123/sodium oleate solution containing the PdCu nanoparticles with an acidic solution of the polymer precursors containing 2,4‐dihydroxybenzoic acid (DA) and hexamethylentetramine (HMT). During the hydrothermal treatment, the polymerization of DA and formaldehyde (generated from HMT) takes place at the surface of the emulsion droplets to generate hollow polymer spheres (HPS, consistent with the results reported in literature^7^). Meanwhile the PdCu nanoparticles in the emulsion droplets are in situ encapsulated in the HPS to obtain PdCu@HPS, which can be converted to PdCu@HCS after pyrolysis at 500 °C under Ar atmosphere. To ensure the comparability with PdCu@HCS, for the synthesis of PdCu/HCS (path B), the PdCu nanoparticles are deposited on the outside surfaces of the presynthesized HPS (without PdCu nanoparticles inside) to obtain PdCu/HPS. Subsequent pyrolysis following the same pyrolysis procedure provides PdCu/HCS. Since carbon‐supported metal nanoparticles often get blocked by carbonaceous residues during pyrolysis,7a before characterization and utilization, the samples of PdCu@HCS and PdCu/HCS are activated by H_2_O_2_ treatment (60 °C for 4 h), followed by reduction with H_2_/Ar (10 %/90 %) at 300 °C for 6 h.

**Figure 1:**
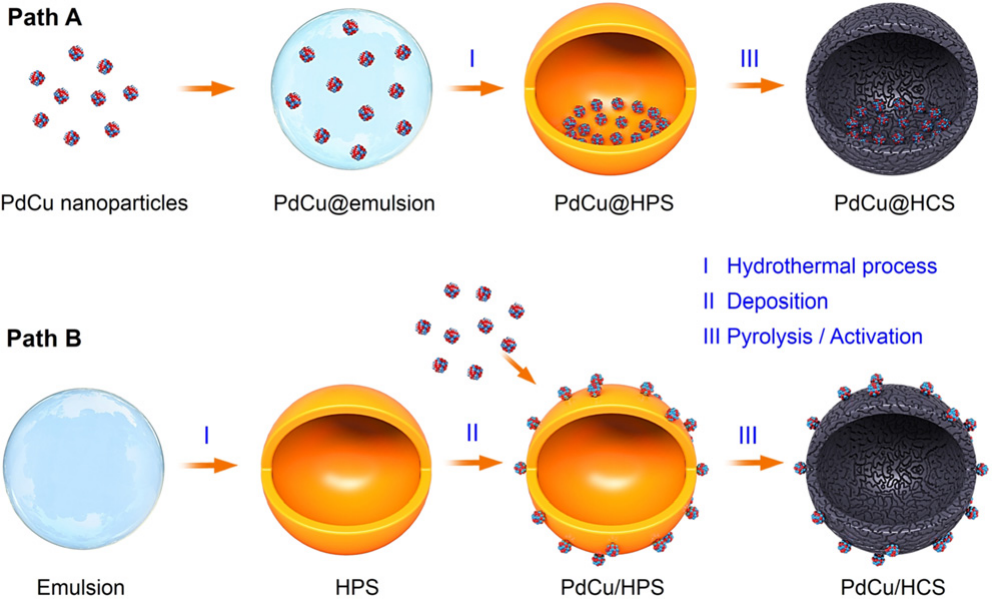
Schematic illustration of the synthesis of PdCu@HCS (path A) and PdCu/HCS (path B).

High‐angle annular dark‐field scanning transmission electron microscopy (HAADF‐STEM) images of PdCu@HCS clearly show that the PdCu nanoparticles (2.7±0.6 nm, Figure S1c,d) are encapsulated in the hollow cores of HCS after pyrolysis and activation (Figure 2 a,b), and no PdCu nanoparticles are observed on the outside surfaces of HCS (Figure S2a,b). The average diameter and shell thickness of PdCu@HCS are 136±23 nm and 16±4 nm, respectively (Figure 2 c). In contrast, the PdCu nanoparticles (2.9±0.5 nm, Figure S1e,f) of PdCu/HCS are uniformly dispersed on the outside surfaces of HCS (Figures 2 d,e and S2c,d). Accordingly, the average diameter and shell thickness of PdCu/HCS are 120±25 nm and 14±4 nm, respectively (Figure 2 f), which are very similar to the HCS structural parameters of PdCu@HCS. In addition, the N_2_ sorption isotherms of PdCu@HCS and PdCu/HCS almost overlap with each other (Figure 2 g), both of which exhibit a steep uptake at relative pressure *p*/*p*_0_<0.01 (indicating the existence of micropores in the carbon shells) and a hysteresis loop at relative pressure closing around *p*/*p*_0_≈0.42 (characteristic of hollow structures).^7^ The pore size distributions of the two nanoreactors are also the same (Figure 2 g, inset) and display similar peaks (mainly centered around 0.60 nm) in the micropore region. The Brunauer–Emmett–Teller (BET) surface areas and the total pore volumes are 509 m^2^ g^−1^ and 1.11 cm^3^ g^−1^ for PdCu@HCS, and 546 m^2^ g^−1^ and 0.89 cm^3^ g^−1^ for PdCu/HCS, respectively (Table S1). These results indicate that the two nanoreactors, PdCu@HCS and PdCu/HCS, exhibit similar microporous carbon shells and hollow structures.

**Figure 2:**
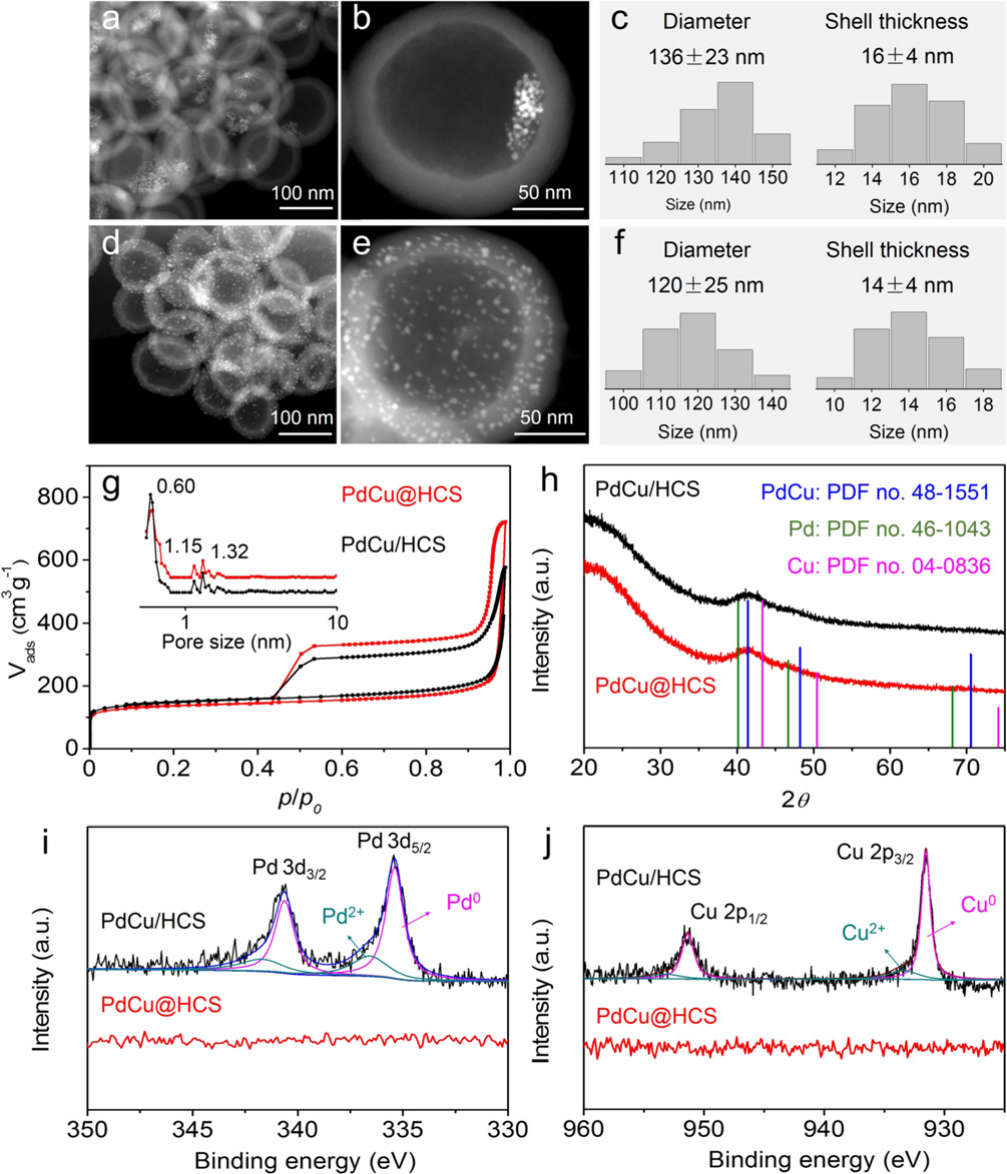
a,b) STEM images, c) the diameter and shell thickness distributions of PdCu@HCS; d,e) STEM images, f) the diameter and shell thickness distributions of PdCu/HCS; g) N_2_ sorption isotherms, h) XRD patterns, i) Pd 3d and j) Cu 2p spectra of PdCu@HCS and PdCu/HCS.

Moreover, the X‐ray diffraction (XRD) patterns of those two nanoreactors show the same diffraction peaks, which can be assigned to the PdCu alloy with face‐centered cubic structure (PDF No. 48‐1551), indicating the PdCu nanoparticles in both of the two nanoreactors have the same crystal structures (Figure 2 h). According to inductively coupled plasma optical emission spectrometry (ICP‐OES), the loadings of Pd and Cu are almost the same for PdCu@HCS (Pd: 3.0 wt %, Cu: 1.9 wt %, Table S1) and PdCu/HCS (Pd: 3.3 wt %, Cu: 1.8 wt %, Table S1), findings that are also in agreement with the thermogravimetric analysis (TGA, Figure S3). Furthermore, the distribution features of PdCu nanoparticles on the PdCu@HCS and PdCu/HCS are surveyed by X‐ray photoelectron spectroscopy (XPS). As expected, no Pd 3d and Cu 2p signals are detected for PdCu@HCS (Figures 2 i,j and S4), indicating that the PdCu nanoparticles are completely encapsulated by the HCS. In contrast, the peaks of Pd 3d and Cu 2p spectra are observed for PdCu/HCS (Figure 2 i,j and S4), where the Pd 3d spectrum is split into four peaks associated with Pd^0^ and Pd^2+^ chemical states,6a, 8 and the Cu 2p spectrum shows peaks that can be assigned to Cu^0^ and Cu^2+^ chemical states.2k The chemical states of Pd^2+^ and Cu^2+^ may be caused by the formation of a passivated layer on the surface of PdCu nanoparticles under air atmosphere. Based on the above analysis, it can be clearly concluded that the nanoreactors of PdCu@HCS and PdCu/HCS have similar porous structures of HCS as well as similar crystal structures of PdCu nanoparticles. The only difference is the spatial location of PdCu nanoparticles: the PdCu nanoparticles are encapsulated inside of the HCS for PdCu@HCS and supported outside of the HCS for PdCu/HCS, respectively. Therefore, the two hollow nanoreactors can be used as ideal models for comparatively investigating the void‐confinement effects induced by the hollow structures.

In order to ensure that the catalytic tests over the two hollow nanoreactors are performed under the same reaction conditions, a simple two‐chamber reaction system was developed to assist in the decoupling of the void‐confinement effects from the influence of reaction conditions, where the two glass tubes are connected with H_2_ balloon through a three‐way tube with valves and placed in a magnetic stirrer water bath (Figures 3 a and S5). In addition, the hydrogenation of styrene (0.42×0.72 nm, according to the structure from Chem3D, Table S2) to ethylbenzene was chosen as a model reaction to evaluate the catalytic behavior of the two nanoreactors. As shown in Figures 3 b and S6, the hydrogenation of styrene occurred over both PdCu@HCS and PdCu/HCS. As expected, but also surprisingly, the hydrogenation rate over PdCu@HCS (9.0 mmol g_cat_^−1^ h^−1^, calculated based on the conversion reached 50 % within a certain time) was obviously faster than that over PdCu/HCS (5.8 mmol g_cat_^−1^ h^−1^); this wasfurther confirmed after several repeated tests (Figure S7a–c). This result provides the experimental evidence that the porous carbon shell of PdCu@HCS can most likely induce an accumulation of styrene molecules and/or hydrogen in the void space,2f leading to an enhanced reaction rate compared with that of PdCu/HCS. It should be mentioned that the reaction curves over PdCu@HCS (I‐repeated) and PdCu/HCS (II‐repeated) are very close to each other (Figure S7d), which may lead to a false conclusion that the catalytic behaviours of the two hollow nanoreactors are similar without obvious void‐confinement effect for PdCu@HCS. This comparison indicates that it is difficult to address the void‐confinement effects when testing the catalytic performances of the two nanoreactors separately even though it is attempted to keep the reaction conditions consistent, since very slight changes in reaction conditions (e.g., H_2_ pressure, reaction temperature and stirring speed) can lead to differences in the catalytic performance. Therefore, it is very necessary to investigate the void‐confinement effects of the hollow nanoreactors in a two‐chamber reactor which is set into a magnetic stirrer water bath to ensure the same reaction conditions in the two chambers (when the same catalyst is used in both chambers, the conversion curves almost overlap with each other, Figure S8).

**Figure 3:**
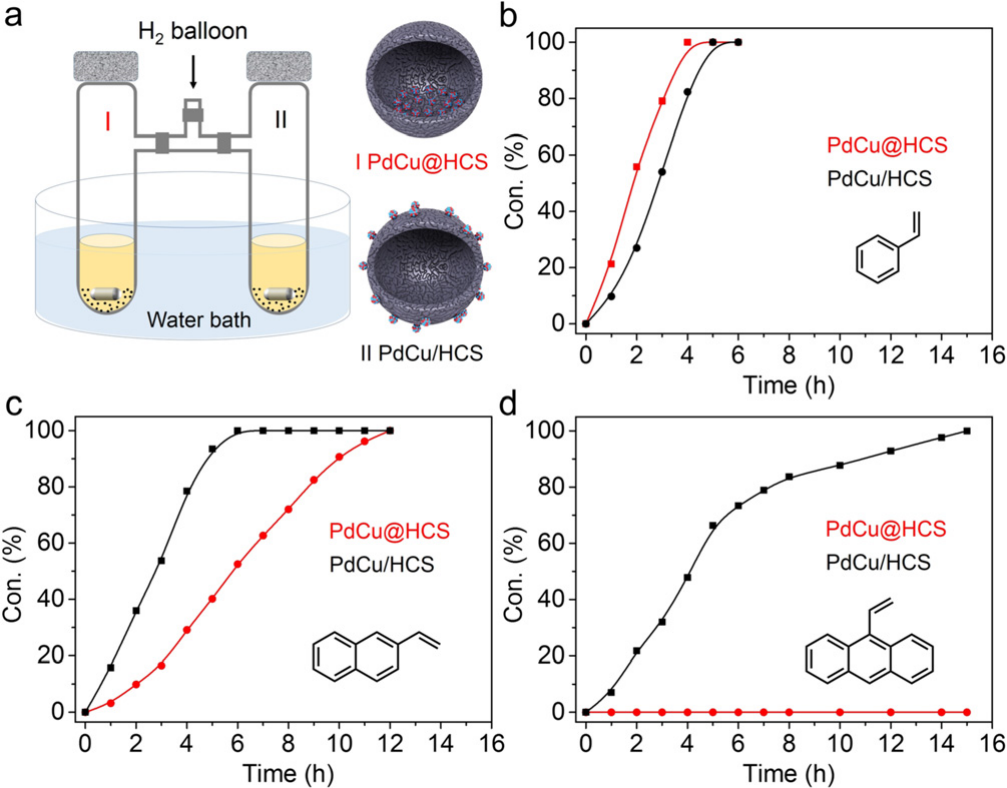
a) Two‐chamber reaction system; hydrogenation of alkenes over PdCu@HCS and PdCu/HCS: b) styrene, c) 2‐vinylnaphthalene, d) 9‐vinylanthracene. Reaction conditions: H_2_ balloon, 25 °C, 30 mg of catalyst, 1 mmol of substrate, 0.5 mmol of dodecane as internal standard, 5 mL of ethanol as solvent. For 9‐vinylanthracene, 0.2 mmol substrate was added.

Moreover, the recycling tests for styrene hydrogenation over the PdCu@HCS and PdCu/HCS were also carried out in the two‐chamber reactor. The catalysts after each run were filtrated and washed with ethanol, followed by drying under vacuum at 50 °C for 8 h. In this way, the two nanoreactors were recycled three times without considerable change in their catalytic performances (Figure S9a–c). The TEM images, N_2_ sorption isotherms, and XRD patterns of the two nanoreactors indicate that their structures were basically unchanged (Figure S9d–g), confirming the high stabilities of the two hollow structured nanoreactors under the mild reaction conditions.

As expected, the effect of mass transfer limitation induced by the HCS on the catalytic process will become gradually more serious with the increasing molecular size of the substrates. As shown in Figure 3 c, the hydrogenation rate of 2‐vinylnaphthalene (0.54×0.92 nm) over PdCu@HCS (2.8 mmol g_cat_^−1^ h^−1^) was much slower than that over PdCu/HCS (6.0 mmol g_cat_^−1^ h^−1^), namely there was no acceleration in 2‐vinylnaphthalene hydrogenation over PdCu@HCS, which can be attributed to the mass transfer limitation for the molecule of 2‐vinylnaphthalene exerted by the microporous carbon shell of PdCu@HCS. For the hydrogenation of 9‐vinylanthracene (0.73×0.91 nm), the conversion over PdCu/HCS was 100 % within 15 h; in contrast, almost no conversion proceeded (<3 %) over PdCu@HCS after 15 h (Figure 3 d), indicating that the nanoreactor of PdCu@HCS restricts the transport of 9‐vinylanthracene through the microporous carbon shells, and then inhibits its hydrogenation.

Based on the above comparative analysis, the nanoreactor of PdCu@HCS with microporous carbon shells (pore size is mainly centered around 0.60 nm, according to N_2_ sorption analysis) can accelerate the hydrogenation of styrene (0.42×0.72 nm) via an accumulation of reactant molecules in the void space, decelerate the hydrogenation of 2‐vinylnaphthalene (0.54×0.92 nm) due to the mass transfer limitation, and inhibit the hydrogenation of 9‐vinylanthracene (0.73×0.91 nm) because of the molecular‐sieving effect. (The hydrogenation processes over the hollow nanoreactors have been modeled based on the literature,^9^ Figure S10.) These studies provide straightforward examples for understanding and estimating the void‐confinement effects of hollow nanoreactors (e.g., accumulation of reactant molecules, mass transfer limitation, molecular‐sieving effect) which are highly related to both the pore size of the shells and the molecular size of the substrates. Notably, when the reaction temperature is increased to 60 °C, the reaction curves for styrene hydrogenation over PdCu@HCS and PdCu/HCS almost overlap with each other (Figure S11), indicating that the void‐confinement effects of PdCu@HCS for accelerating hydrogenation of small molecules (such as styrene) are temperature dependent.

Next, the hydrogenation of phenylacetylene (0.42×0.74 nm) over the two nanoreactors was also investigated in the two‐chamber reactor. As shown in Figure 4 a, the conversion of phenylacetylene over PdCu/HCS reached 100 % after reaction for 5 h with selectivity towards styrene higher than 82 % (Figure 4 b); after the complete consumption of phenylacetylene, the styrene was quickly converted into ethylbenzene (Figure 4 b). However, over PdCu@HCS full conversion of phenylacetylene was reached after reaction for 7 h with a styrene selectivity of only 32 % (Figure 4 a,b). These results indicate that the conversion of styrene over PdCu@HCS is enhanced compared with that over PdCu/HCS (Figure S12). Since the structures of the PdCu nanoparticles in both PdCu@HCS and PdCu/HCS are similar, the difference in the catalytic performance of the two nanoreactors should be attributed to the void‐confinement effects of PdCu@HCS, which can result in relatively high local concentration and long retention time of the in situ generated intermediate of styrene in the void space, and therefore promote the hydrogenation of styrene. Accordingly, the slower hydrogenation rate of phenylacetylene over PdCu@HCS (4.2 mmol g_cat_^−1^ h^−1^) compared with PdCu/HCS (5.6 mmol g_cat_^−1^ h^−1^) can be ascribed to the competitive hydrogenation of phenylacetylene and styrene in the void space. Similar catalytic performances were found for the hydrogenation of 1‐ethynylnaphthalene (0.66×0.74 nm) and 2‐octyn‐1‐ol over the two nanoreactors (Figures S13a,b and S14a,b). These results confirm that the void space of the hollow nanoreactors, which provides a unique microenvironment for reactions, can alter the hydrodynamics of reactants or intermediates, and correspondingly change the catalytic properties during reactions. In addition, the molecular‐sieving effect of PdCu@HCS was again demonstrated by hydrogenation of 9‐ethynylphenanthrene (0.78×0.90 nm), where almost no conversion proceeded (Figure S13c,d). Moreover, the reaction curves for hydrogenations of 2‐octyn‐1‐ol and phenylacetylene also tended to be the same when the reaction temperature was increased to 60 °C (Figures S14c,d and S15), further indicating that the void‐confinement effects of PdCu@HCS are temperature dependent for the hydrogenation of small molecules.

**Figure 4:**
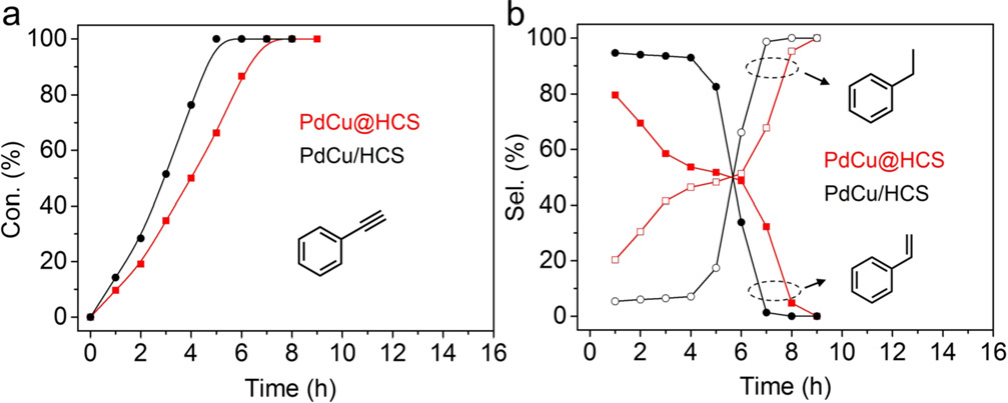
Conversion (a) and selectivity (b) for the hydrogenation of phenylacetylene over PdCu@HCS and PdCu/HCS. Reaction conditions: H_2_ balloon, 25 °C, 30 mg of catalyst, 1 mmol of substrate, 0.5 mmol of dodecane as internal standard, 5 mL of ethanol as solvent.

Based on the catalytic properties of the hollow nanoreactor of PdCu@HCS, efficient synthesis of important organic compounds can be achieved. Imines are an important class of chemicals that can be used as intermediates in various reactions (e.g., condensation, cycloaddition, and reduction reactions) for the generation of pharmaceuticals, agricultural chemicals, and other useful fine chemicals.^10^ An attractive way to synthesize imines is via cascade reductive coupling of nitro compounds with carbonyl compounds. In this process, however, preventing the over‐hydrogenation of imines to amines remains an issue. Here, nitrobenzene (0.43×0.59 nm) and phenanthrene‐9‐carboxaldehyde (0.68×0.90 nm) are chosen as precursors for synthesizing the target imine, *N*‐(9‐phenanthrenylmethylene)benzenamine, using PdCu@HCS as the catalyst (Figure 5 a). In the first step, the nitrobenzene can quickly diffuse into the void space of PdCu@HCS (as inferred from the hydrogenation of styrene and phenylacetylene, Figures 3 b and 4 a), and convert into aniline through a reductive process. The formed aniline will then condense with the phenanthrene‐9‐carboxaldehyde outside of the hollow nanoreactor to generate the target imine (experiment indicates that the condensation reaction can proceed without catalysts). In fact, the molecular size of phenanthrene‐9‐carboxaldehyde is similar to the molecules of 9‐vinylanthracene and 9‐ethynylphenanthrene, which cannot diffuse through the carbon shells of PdCu@HCS. Therefore, it can be expected that the over‐reduction of the target imine to amine can be avoided through the molecular‐sieving effect. As shown in Figure 5 b, the conversion of nitrobenzene over the PdCu@HCS reached 100 % after reaction for 3 h, and imine selectivity was indeed higher than 99 % (Figure S16). In contrast, the imine was completely converted into amine when PdCu/HCS was used as the catalyst (Figure S17). These results indicate that the specific imines as well as other organic compounds can be efficiently and selectively produced by utilizing the unique features of the hollow nanoreactors.

**Figure 5:**
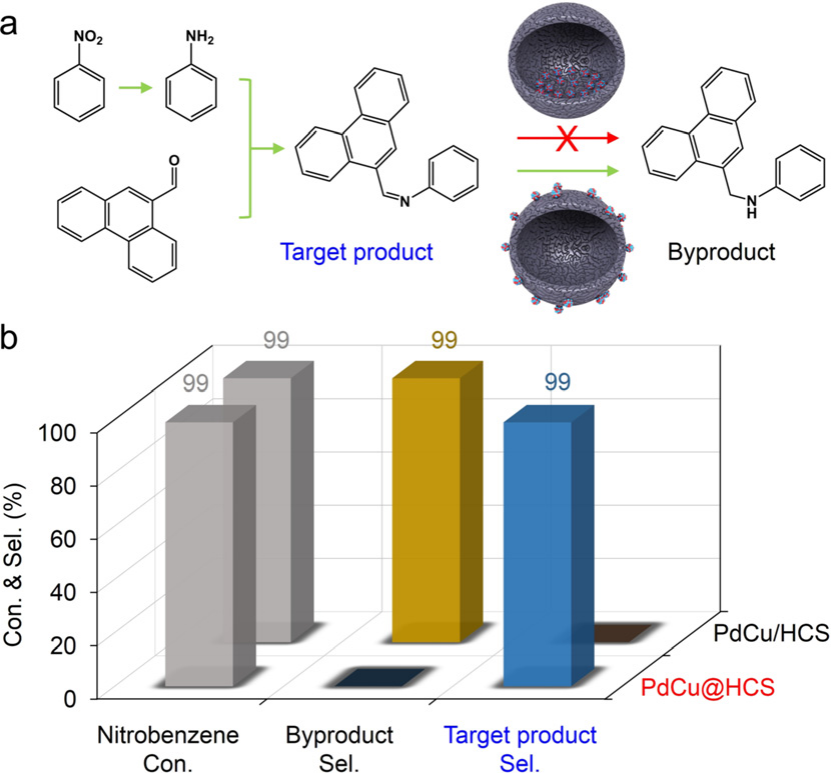
a) Scheme of the cascade reductive coupling of nitroarene with a carbonyl compound; b) conversion and selectivity diagrams of the cascade reaction over PdCu@HCS and PdCu/HCS.

In conclusion, we have developed a general strategy to synthesize a pair of differently loaded hollow nanoreactors (PdCu@HCS and PdCu/HCS). Based on the two comparative nanoreactors, the void‐confinement effects of hollow nanoreactors in liquid‐phase hydrogenation have been extensively investigated in the two‐chamber reactor. It was found that the PdCu@HCS can accelerate the hydrogenation of styrene via an accumulation of reactant molecules, decelerate the hydrogenation of 2‐vinylnaphthalene due to the mass transfer limitation, and inhibit the hydrogenation of 9‐vinylanthracene because of the molecular‐sieving effect. In addition, the void space of the PdCu@HCS can alter the hydrodynamics of the intermediate, and correspondingly change the catalytic selectivity during hydrogenation of small alkynes. Moreover, a specific imine has been selectively produced over the PdCu@HCS by utilizing the shape‐selective catalysis principle. These studies provide straightforward examples for clearly understanding and estimating the void‐confinement effects of the hollow nanoreactors. It is expected that the synthesis strategy can be extended to other metal systems, and meanwhile the HCS structures (diameters, shell thicknesses, and porous structures) as well as the loading amount of metal nanoparticles can be tailored by fine‐tuning the synthetic parameters. Therefore, various pairs of hollow nanoreactors can be designed and employed as ideal models for study of catalytic mechanisms in various reactions, and thus guide the design of efficient catalysts for specific chemical transformations and the development of nanoreactor reaction engineering.

## Conflict of interest

The authors declare no conflict of interest.

## Supporting information

As a service to our authors and readers, this journal provides supporting information supplied by the authors. Such materials are peer reviewed and may be re‐organized for online delivery, but are not copy‐edited or typeset. Technical support issues arising from supporting information (other than missing files) should be addressed to the authors.

Supplementary
