# Supplementary material for: Hollow Carbon Sphere Nanoreactors Loaded with PdCu Nanoparticles: Void‐Confinement Effects in Liquid‐Phase Hydrogenations
Source: Angew Chem Int Ed Engl. 2020 Aug 18;59(42):18374–9. doi: 10.1002/anie.202007297 (PMC7590117; doi:10.1002/anie.202007297)
Supplement: Supplementary file 1 — Supplementary [file ANIE-59-18374-s001.pdf]

Angewandte Chemie
Eine Zeitschrift der Gesellschaft Deutscher Chemiker

Supporting Information

Hollow Carbon Sphere Nanoreactors Loaded with PdCu Nanoparticles: Void-Confinement Effects in Liquid-Phase Hydrogenations

Chao Dong[+], Qun Yu[+], Run-Ping Ye, Panpan Su, Jian Liu, and Guang-Hui Wang**

anie_202007297_sm_miscellaneous_information.pdf

Experimental Procedures

Materials. Pluronic P123, 2,4-dihydroxybenzoic (DA, 97%) and hexamethylenetetramine (HMT, 99%) were purchased from Sigma-Aldrich. Sodium oleate (SO, 97%), trioctylamine (TOA, 97%), copper acetylacetonate ($Cu(acac)_2$, 97%) and palladium acetylacetonate ($Pd(acac)_2$, 99%), phenylacetylene (97%) and phenanthrene-9-carboxaldehyde (98%) were purchased from Aladdin. Nitrobenzene (99%), styrene (AR), dodecane (99%), oleic acid (OA, AR), hydrogen peroxide (H_2O_2, 30%) and ethanol absolute (⩾99.7%) were purchased from Sinopharm Chemical Reagent Co.,Ltd. 1-Ethynylnaphthalene was purchased from leyan Co., Ltd. 9-Ethynylphenanthrene was purchased from Tokyo Chemical Industry Co., Ltd. (TCI). 2-vinylnapthalene (>95%) and 9-vinylanthracene (97%) was purchased from Energy Chemical. All reagents were used as received without further purification.

Synthesis of PdCu nanoparticles. PdCu nanoparticles were prepared by a solvothermal method reported previously[1] with a small modification. Typically, 0.05 mmol $Pd(acac)_2$, 0.05 mmol $Cu(acac)_2$, 10 mL TOA, and 1 mL OA were mixed in a round-bottom flask to form a homogeneous mixture. Then, the mixture was heated at 200 ºC for 30 min with stirring under argon atmosphere. After cooling to room temperature, 30 mL of ethanol was added into the reaction solution, and the resulting colloidal product of PdCu nanoparticles was collected by centrifugation (14000 rpm, 3 min). After washing two times with an ethanol/hexane mixture, the collected product was re-dispersed in 870 μL of hexane for further use.

Sysnthesis of PdCu@HCS and PdCu/HCS.

For the synthesis of PdCu@HCS: one half of the above hexane solution (435 μL) containing PdCu nanoparticles was added into 2 mL of water containing 0.24 mmol of SO. The mixture was ultrasonicated for 45 min, and then evaporated the hexane at 60 ºC to get a clear colloidal solution (PdCu/SO solution). Then, 4 mL of water containing 0.0075 mmol P123 was added into the PdCu/SO solution and ultrasonicated for 10 min. The obtained PdCu/SO/P123 colloidal solution was added into 74 mL of aqueous solution containing 1.2 mmol of DA and 0.5 mmol of HMT under slow stirring. The as-obtained emulsion solution containing the PdCu nanoparticles was transferred into a teflon-lined stainless steel autoclave of 120 mL capacity, sealed and treated at 160 ºC for 4 h. The product (denoted as PdCu@HPS) was collected by centrifugation, washed with deionized water for three times, and finally dried at 50 ºC under vacuum for 8 h. Finally, PdCu@HCS was obtained after pyrolysis at 500 ºC under Ar atmosphere. The pyrolysis procedure under argon was as follows: the sample was heated to 400 ºC at a ramping rate of 2 ºC min^{-1} and maintained at this temperature for 3 h, and then heated to 500 ºC at a ramping rate of 1 ºC min^{-1} and maintained at this temperature for 2 h.

For the synthesis of PdCu/HCS: 2 mL of water containing 0.24 mmol of SO and 4 mL of water containing 0.0075 mmol P123 were mixed to form the SO/P123 solution. Then, the solution was added into 74 mL of aqueous solution containing 1.2 mmol of DA and 0.5 mmol of HMT under slow stirring. The as-obtained emulsion solution was transferred into a Teflon-lined stainless steel autoclave of 120 mL capacity, sealed and treated at 160 ºC for 4 h. The product (denoted as HPS) was collected by centrifugation, washed with ethanol and hexane, and then re-dispersed in 10 mL of hexane. Next, the other half of the hexane solution (435 μL) containing PdCu nanoparticles was added into the hexane solution containing HPS. The mixture was ultrasonicated for 20 min, and then stirred for 2 h (ensuring uniform distribution of PdCu nanoparticles). After removal the hexane by evaporation (ensuring complete loading of PdCu nanoparticles), the product (denoted as PdCu/HPS) was obtained. At last, the PdCu/HCS was obtained after pyrolysis using the same procedure with that of PdCu@HCS.

For H_2O_2/H_2 treatment of PdCu@HCS and PdCu/HCS: 100 mg of the sample was dispersed into 10 mL of H_2O_2 (30% w/w), treated at 60 ºC for 4 h in a round-bottom flask with a glass condenser. Then, the sample was washed with deionized water, dried at 50 ºC under vacuum, and followed by the reduction under H_2/Ar atmosphere at 300 ºC for 6 h.

Hydrogenation of alkenes and alkynes. To ensure that the catalytic tests over the PdCu@HCS and PdCu/HCS were performed under the same reaction conditions, a simple two-chamber reaction system was designed, where the two glass tubes (10 mL capacity) were connected with H_2 balloon through a three-way tube with valves and placed in a magnetic stirrer water bath. During catalytic tests, one glass tube was loaded with the PdCu@HCS, and the other one was loaded with the PdCu/HCS. Typically, in each glass tube, 1 mmol substrate, 0.5 mmol dodecane (internal standard), 30 mg catalyst and 5 mL of ethanol were added. For 9-vinylanthracene, 0.2 mmol substrate and 0.1 mmol dodecane were added. Then, the air in the two-chamber reaction system was removed by vaccum, and the H_2 was filled into the system via a hydrogen balloon. The reactions were performed at 25 ºC under stirring at 300 rpm. A small volume of sample (~0.1 mL) in each glass tube was periodically withdrawn and analyzed by GC (Agilent 7890B), GC-MS (Agilent 7890B-5977B) or ^{1}H NMR spectroscopy (AVANCE-III 600 MHz).

Cascade reductive imination of nitrobenzene. Typically, 0.5 mmol of nitrobenzene, 0.75 mmol of phenanthrene-9-carboxaldehyde, 0.5 mmol dodecane (internal standard), 50 mg of catalyst and 5 mL of toluene were added into a glass inset, transferred in a stainless-steel autoclave reactor (50 mL), and then sealed and purged with H_2 several times. The reaction was performed at 80 ºC with initial H_2 pressure of 10 bar under stirring at 300 rpm. After reaction for 3 h, the reactor was cooled down to room temperature. The solution was filtered and analyzed by ^{1}H and ^{13}C NMR spectroscopy (AVANCE-III 600 MHz). The conversion was defined as the mole ratio of converted nitrobenzene to initial nitrobenzene. The selectivity of imines based on nitrobenzene was calculated as follow:

$$s = \frac{Imine\ in\ product}{Imine\ in\ product + Amine\ in\ product} \times 100$$

Characterization. Transmission electron microscopy (TEM) was carried out with Hitachi-7650 microscopes with an acceleration voltage of 100 kV. High-resolution transmission electron microscope (HRTEM), high-angle annular dark field scanning transmission electron microscope (HAADF-STEM) and element mappings were carried out with FEI Talos F200S instrument with EDX and STEM attachments at an acceleration voltage of 200 kV. Scanning electron microscope (SEM) and scanning transmission electron microscopy

(STEM) analyses were carried out with HITACHI S-5500 microscopes. Based on the TEM images, the size distributions were determined by counting ~200 hollow spheres (or PdCu nanoparticles) and measuring their diameter and shell thickness (or particle size) using the software of Nano Measurer 1.2. Powder X-ray diffraction (XRD) was performed on a Bruker D8 Advance X-ray diffractometer with Cu Kα radiation (λ=1.5406 Å) at a voltage of 40 kV and a current of 40 mA. X-ray photoelectron spectroscopy (XPS) measurements were conducted on a Thermo ESCALAB 250Xi system with monochromatic Al Kα as the excitation source. Palladium and copper contents of the samples was determined using an inductively coupled plasma-optical emission spectrophotometer (ICP-OES, 730 Series, Agilent, USA). The thermogravimetric analysis (SDT Q600 TA Instruments Co., Ltd) under air was conducted with a heating rate of 10 °C min^{-1}. Nitrogen sorption isotherms were measured with a Micromeritics ASAP 3020 instrument at 77 K. Prior to the measurements, the sample was degassed at 200 °C for 6 h. The specific surface areas and pore size distribution curves were calculated from the adsorption branch data using the Brunauer-Emmett-Teller (BET) method and the non-local density functional theory (NLDFT), respectively.

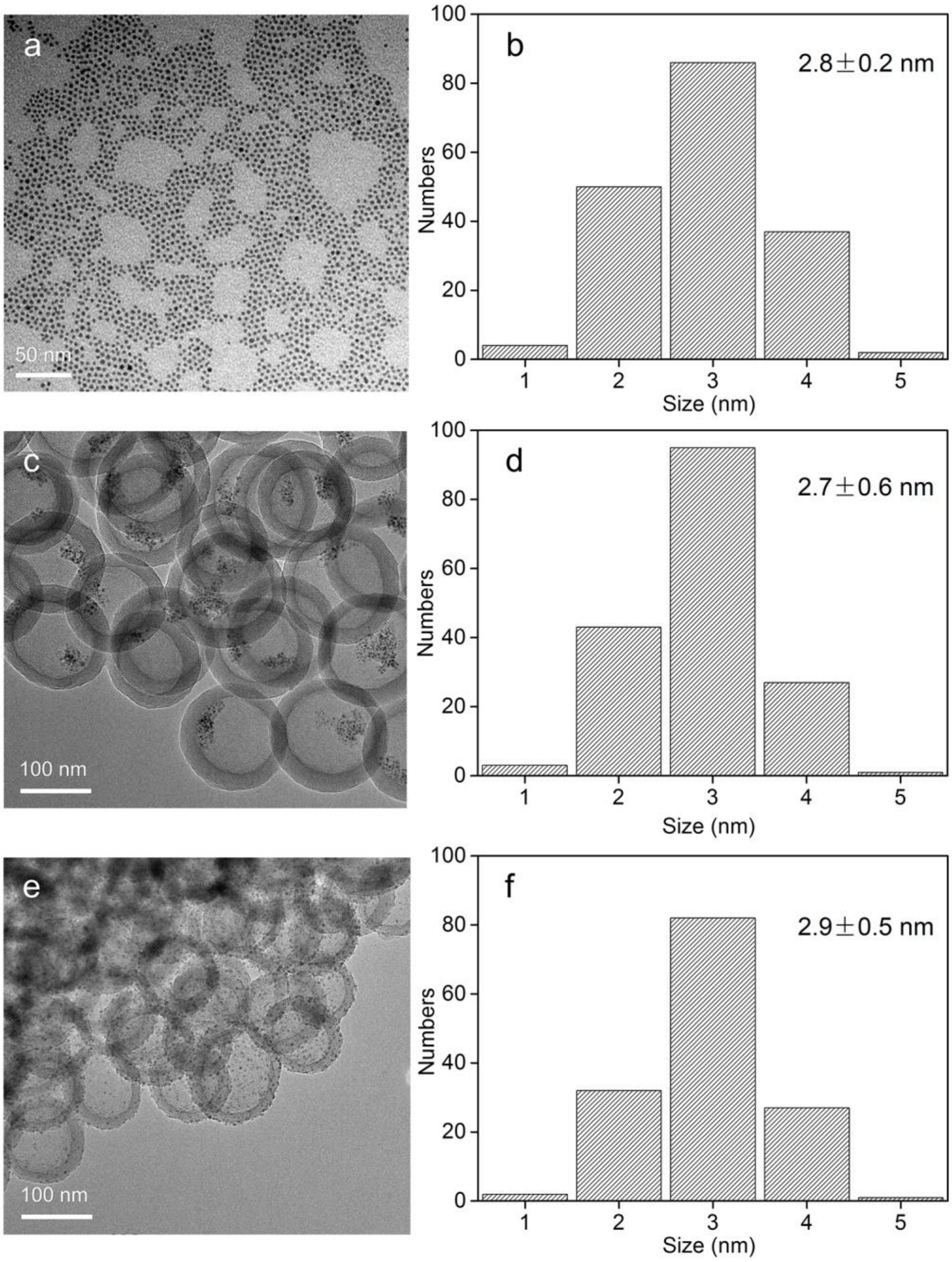

Figure S1. TEM images and the size distributions of PdCu nanoparticles: (a,b) the presynthesized PdCu nanoparticles, (c,d) PdCu@HCS and (e,f) PdCu/HCS.

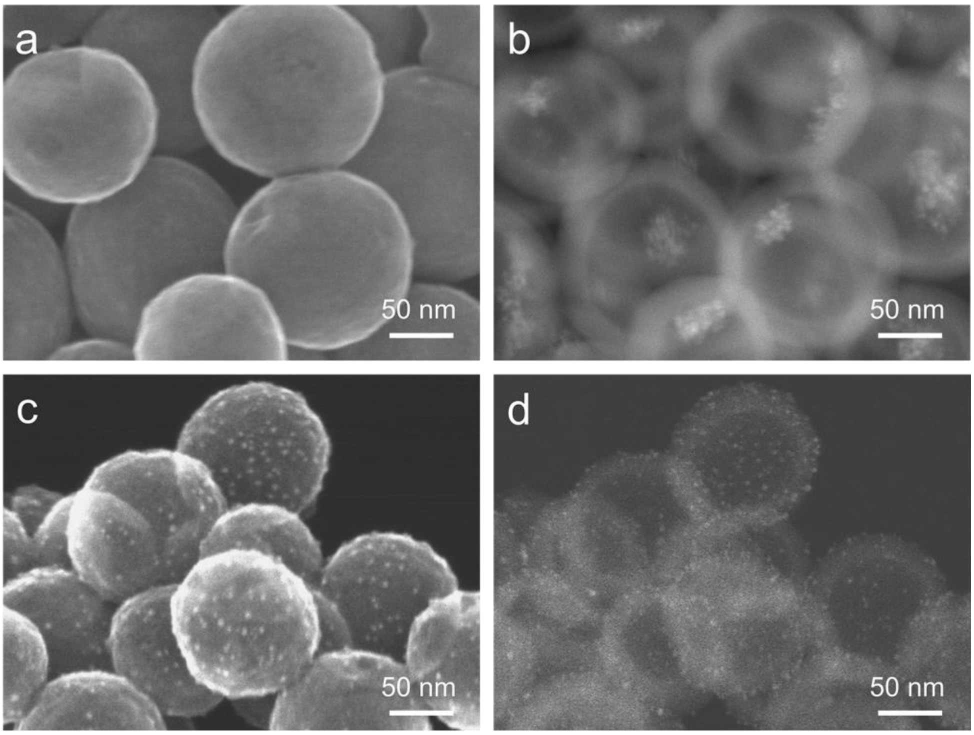

Figure S2. SEM and STEM images of (a,b) PdCu@HCS and (c,d) PdCu/HCS.

Table S1 Textural parameters and metal loadings (determined by ICP-OES) of PdCu@HCS and PdCu/HCS.

Sample	S_{BET} ($m^2 \cdot g^{-1}$)	S_{micro} ($m^2 \cdot g^{-1}$)	V_{total} ($cm^3 \cdot g^{-1}$)	V_{micro} ($cm^3 \cdot g^{-1}$)	Metal loading	
					Pd (wt.%)	Cu (wt.%)
PdCu@HCS	509	429	1.11	0.18	3.0	1.9
PdCu/HCS	546	457	0.89	0.20	3.3	1.8

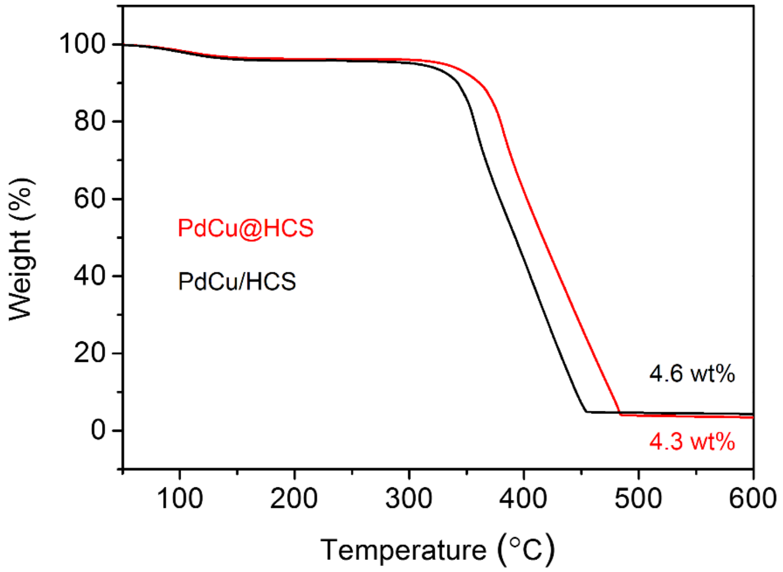

Figure S3. TG curves of PdCu@HCS and PdCu/HCS under air flow.

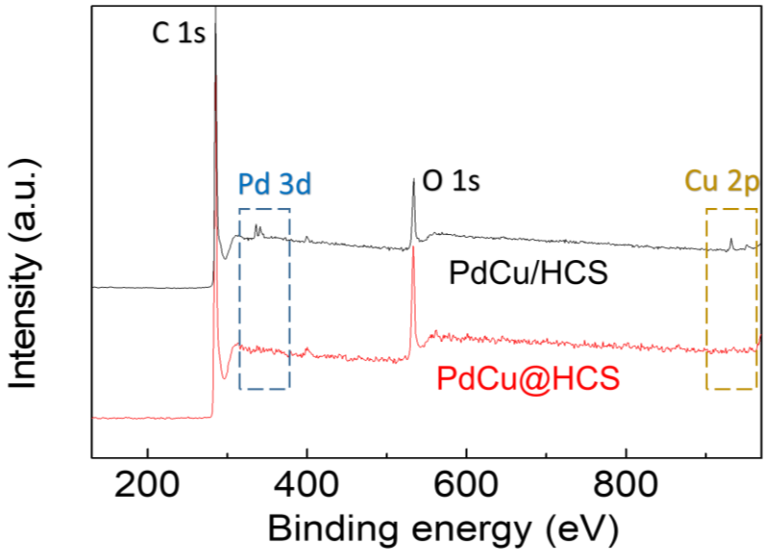

Figure S4. XPS spectra of PdCu@HCS and PdCu/HCS.

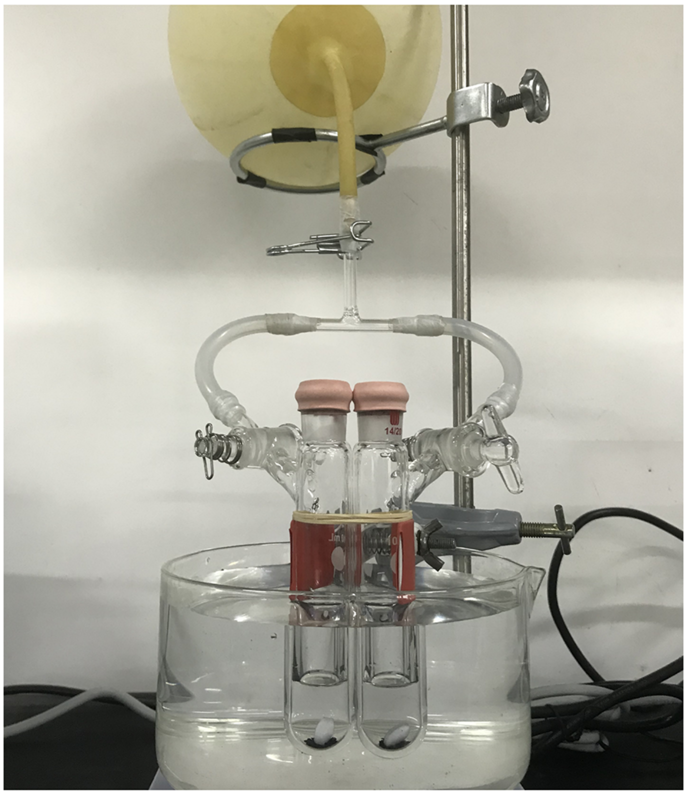

Figure S5. The photograph of the two-chamber reaction system.

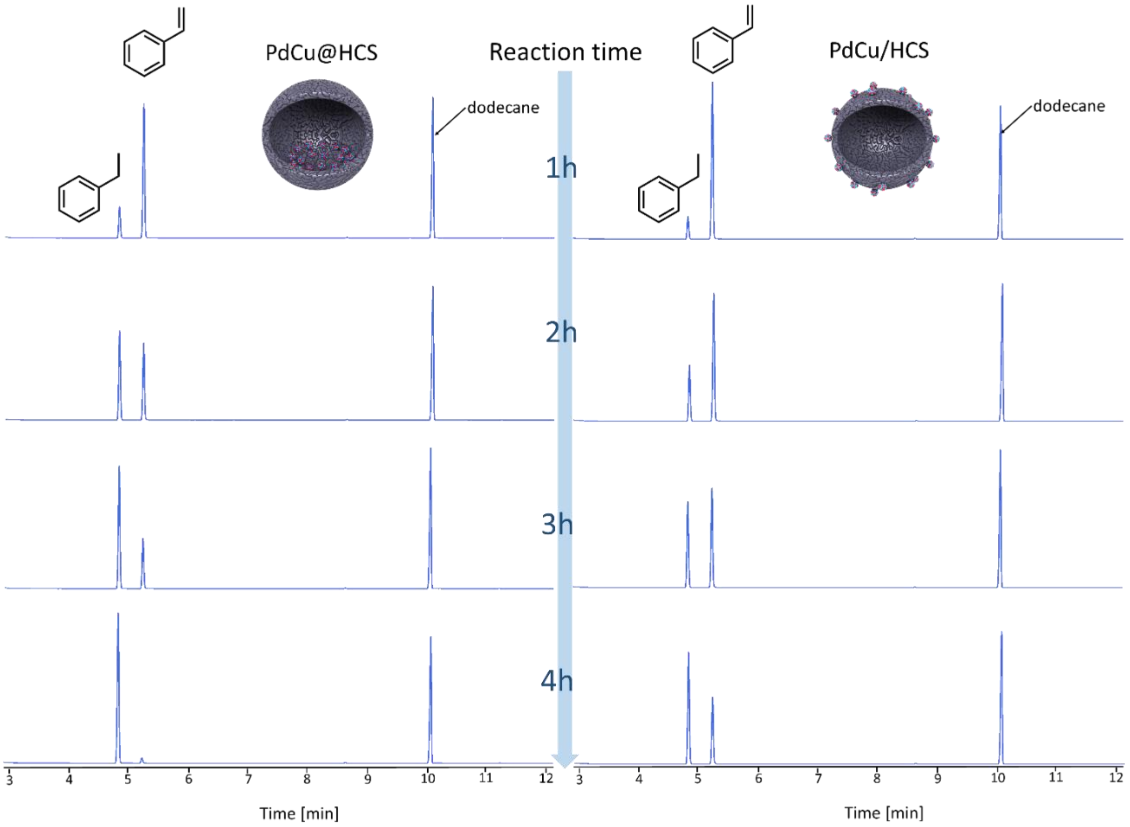

Figure S6. GC analysis (Agilent 7890B) of the products of styrene hydrogenation over PdCu@HCS and PdCu/HCS. Reaction conditions: H_2 balloon, 25 ºC, 30 mg of catalyst, 1 mmol of substrate, 0.5 mmol of dodecane as internal standard, 5 mL of ethanol as solvent.

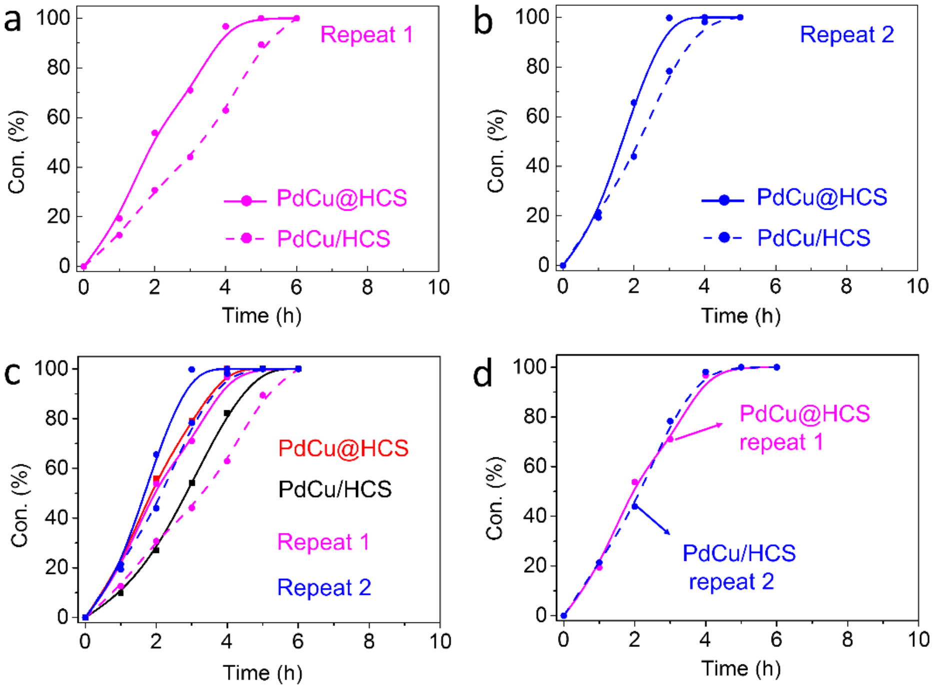

Figure S7. Repeat tests of styrene hydrogenation over PdCu@HCS and PdCu/HCS. For each repeat test, the PdCu@HCS and PdCu/HCS were newly-synthesized. Reaction conditions: H_2 balloon, 25 ºC, 30 mg of catalyst, 1 mmol of substrate, 0.5 mmol of dodecane as internal standard, 5 mL of ethanol as solvent.

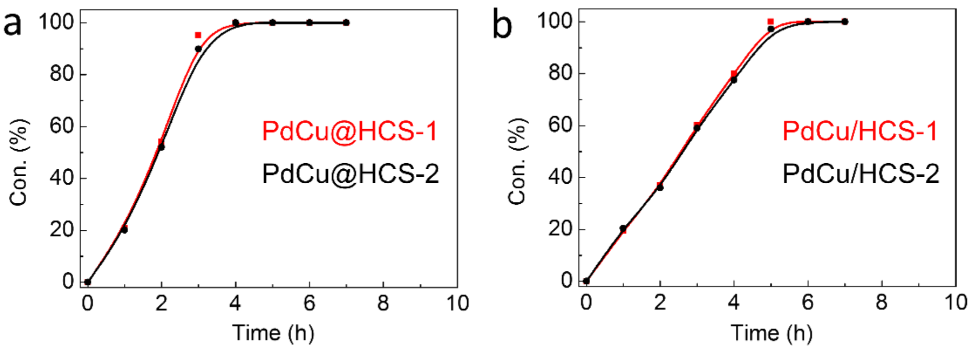

Figure S8. Styrene hydrogenation in the two-chamber reactor: (a) PdCu@HCS as catalyst in both chambers; (b) PdCu/HCS as catalyst in both chambers. Reaction conditions: H_2 balloon, 25 ºC, 30 mg of catalyst, 1 mmol of substrate, 0.5 mmol of dodecane as internal standard, 5 mL of ethanol as solvent.

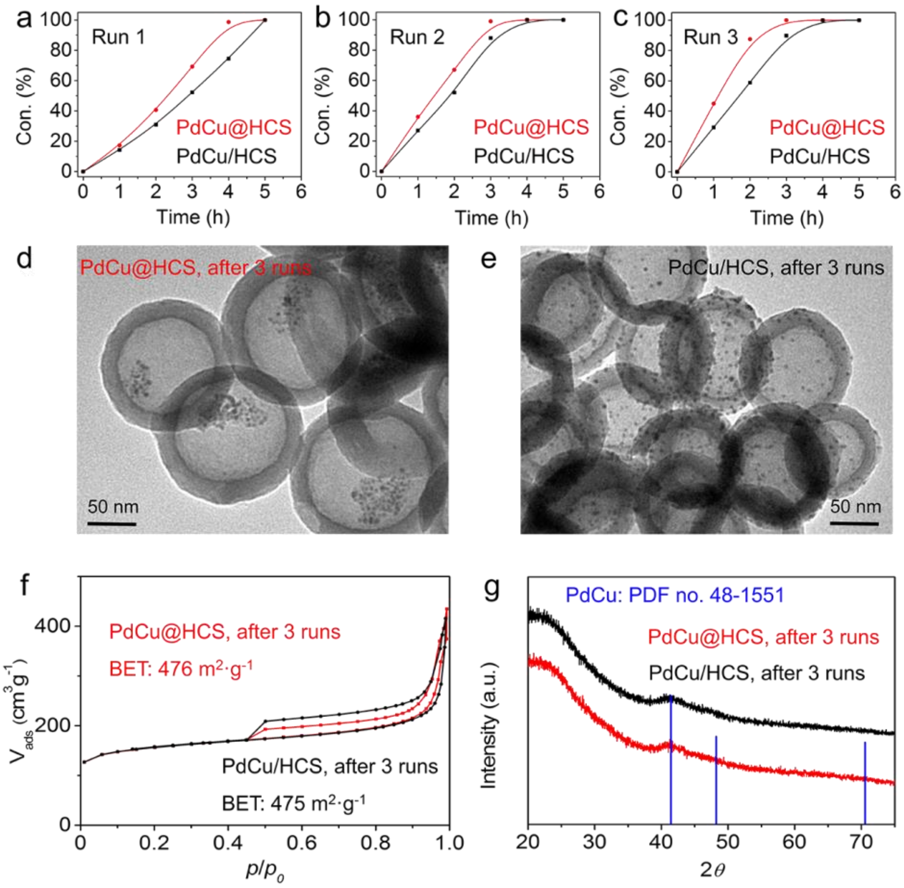

Figure S9. (a-c) Catalytic recycling results for styrene hydrogenation over PdCu@HCS and PdCu/HCS. Reaction conditions: H_2 balloon, 25 ºC, 30 mg of catalyst, 1 mmol of substrate, 0.5 mmol of dodecane as internal standard, 5 mL of ethanol as solvent. (d,e) TEM images, (f) N_2 sorption isotherms and (g) XRD patterns of PdCu@HCS and PdCu/HCS after recycling 3 times.

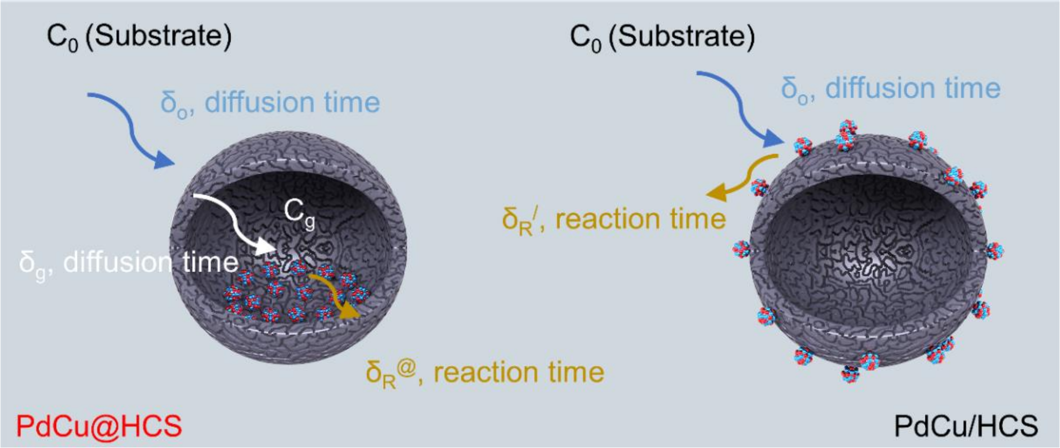

Figure S10. Schematic illustration for hydrogenation process over the hollow nanoreactors of PdCu@HCS and PdCu/HCS.

In our system, H_2 is in large excess with respect to the substrate, which is assumed to transfer through the microporous shells of the hollow nanoreactors with larger diffusion rate than the substrate. Therefore, the hydrogenation of vinyl compounds can be treated as pseudounimolecular reaction, and the total reaction rate k_t can be written as:[2,3]

$k_t^{-1} = k_R^{-1} + k_D^{-1}$ or $\delta_t = \delta_R + \delta_D$

where k_R and k_D are the surface reaction rate and diffusion rate; the times δ_t, δ_R and δ_D are the reciprocals of the corresponding rates.

δ_D can be further split into two different contributions (Figure S8): the time to arrive from the bulk solution to the outside surface of hollow nanoreactor, δ_o, that to cross the shell to get to the surface of the nanoparticles δ_g. Therefore, the reaction time over PdCu@HCS and PdCu/HCS can be written as:

$\delta_t^@ = \delta_o + \delta_g + \delta_R^@$ (PdCu@HCS)
$\delta_t^/ = \delta_o + \delta_R^/$ (PdCu/HCS)

When using styrene as substrate,

$\delta_t^@ = \delta_o + \delta_g + \delta_R^@ < \delta_t^/ = \delta_o + \delta_R^/$ (Figure 3b); $\rightarrow \delta_R^@ << \delta_R^/$; namely $k_R^@ >> k_R^/$

Since $k_R = K \cdot C(\text{styrene})^m \cdot C(H_2)^n$, if $k_R^@ >> k_R^/$, the concentrations of C_g(styrene) and/or $C_g(H_2)$ in the void space of PdCu@HCS should be higher than that outside of the shells. This indirectly indicates the hollow nanoreactor of PdCu@HCS can induce the accumulation of reactant molecules in the void space and then accelerate the reaction rate. In addition, it also indicates, to some extent, that the influence of diffusion time δ_g of styrene on the total reaction rate is probably negligible. Therefore, the reaction for styrene hydrogenation over PdCu@HCS is surface controlled.

When using 2-vinylnaphthalene as substrate,

$\delta_t^@ = \delta_o + \delta_g + \delta_R^@ > \delta_t^/ = \delta_o + \delta_R^/$ (Figure 3c); $\rightarrow \delta_g + \delta_R^@ > \delta_R^/$;

In this case, the H_2 still could be enriched in the void space of PdCu@HCS. However, the diffusion of 2-vinylnaphthalene is limited by the microporous shell of PdCu@HCS due to its larger molecular size (or the stronger interaction with the pore walls of PdCu@HCS)

compared with that of styrene, resulting in the increase of diffusion time δ_g which is comparable with the reaction time $\delta_R^@$. Therefore, the reaction for 2-vinylnaphthalene hydrogenation over PdCu@HCS is termed diffusion influenced.

When using 9-vinylanthracene as substrate, no reaction proceeds.

$\delta_t^@ = \delta_o + \delta_g + \delta_R^@ \rightarrow \infty$ (Figure 3d); namely $\delta_g \rightarrow \infty$

In this case, the molecular size of 9-vinylanthracene is too large to transfer through the microporous shell of PdCu@HCS. Therefore, the reaction for 9-vinylanthracene hydrogenation over PdCu@HCS is inhibited.

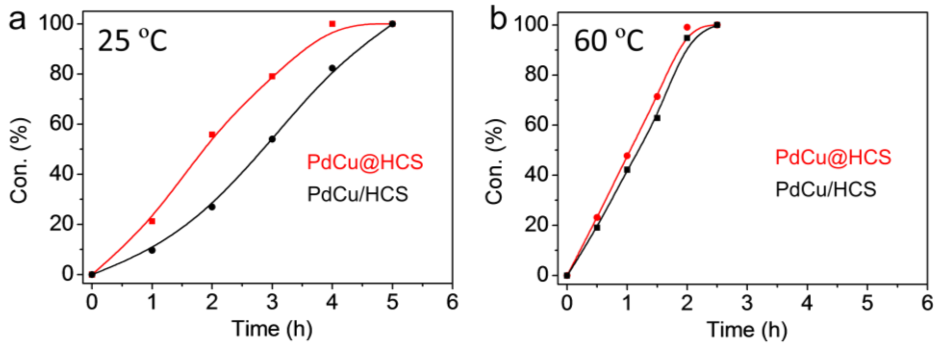

Figure S11. Styrene hydrogenation over PdCu@HCS and PdCu/HCS at (a) 25 ºC and (b) 60 ºC. Reaction conditions: H_2 balloon, 30 mg of catalyst, 1 mmol of substrate, 0.5 mmol of dodecane as internal standard, 5 mL of ethanol as solvent.

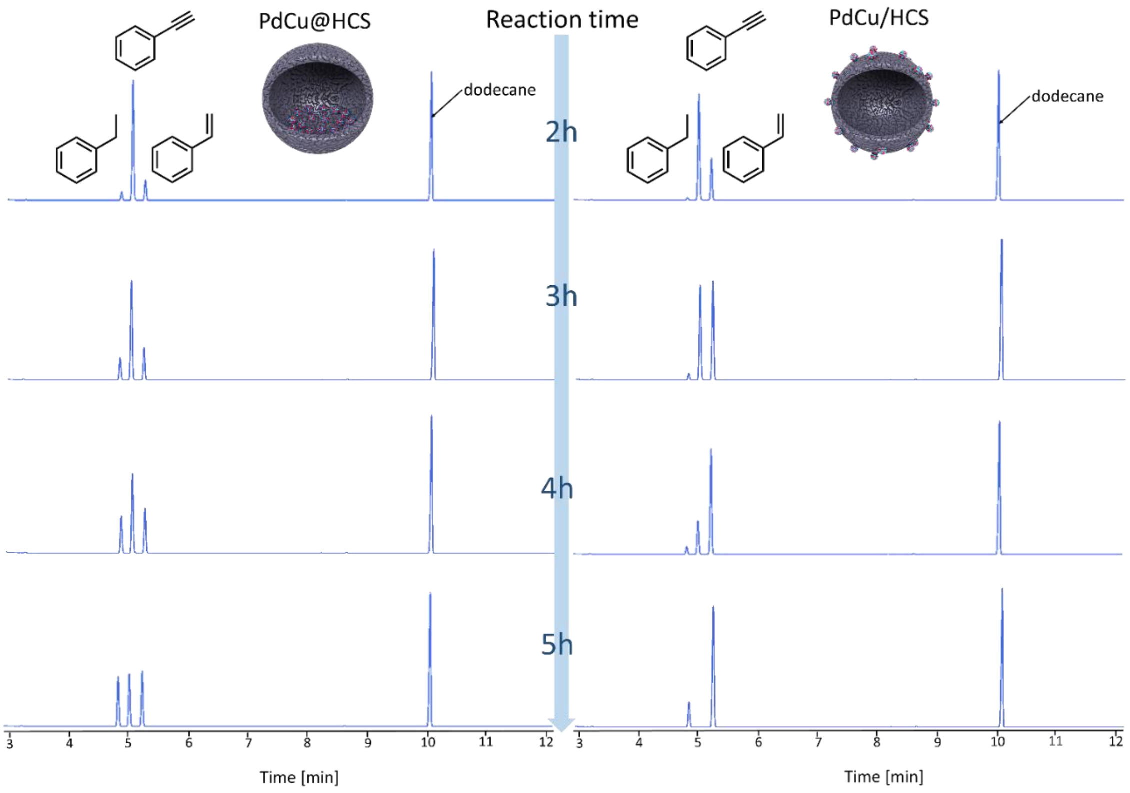

Figure S12. GC analysis (Agilent 7890B) of the products of phenylacetylene hydrogenation over PdCu@HCS and PdCu/HCS. Reaction conditions: H_2 balloon, 25 ºC, 30 mg of catalyst, 1 mmol of substrate, 0.5 mmol of dodecane as internal standard, 5 mL of ethanol as solvent.

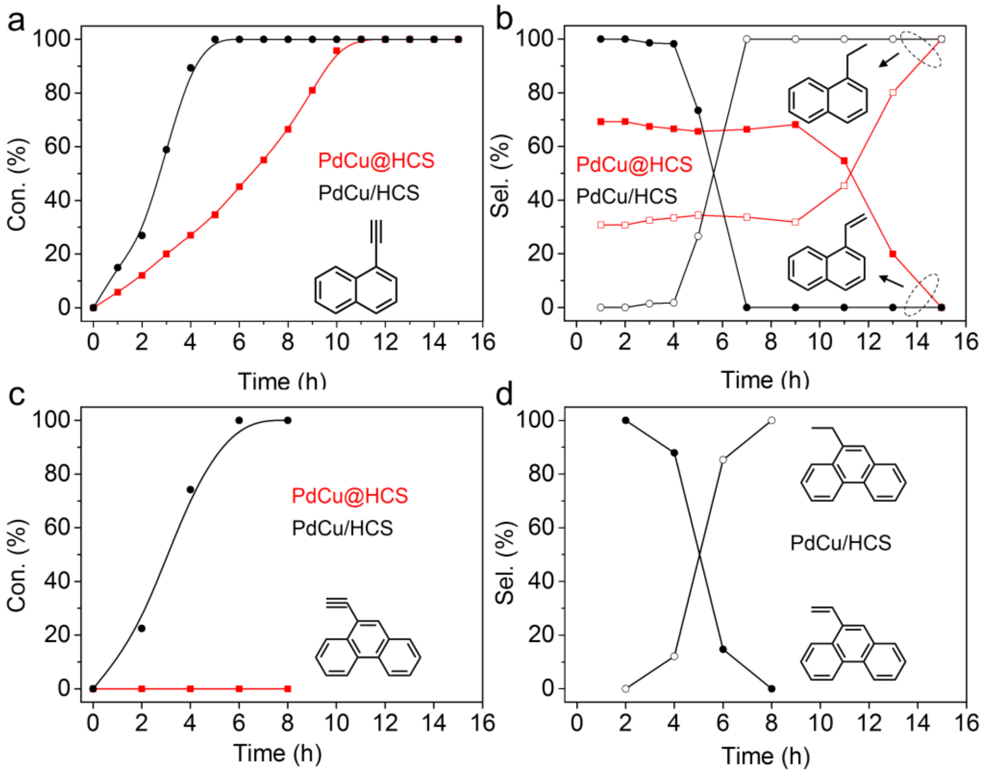

Figure S13. Alkynes hydrogenation over PdCu@HCS and PdCu/HCS: (a,b) 1-ethynylnaphthalene and (c,d) 9-ethynylphenanthrene. Reaction conditions: H_2 balloon, 25 ºC, 30 mg of catalyst, 1 mmol of substrate, 0.5 mmol of dodecane as internal standard, 5 ml of ethanol as solvent.

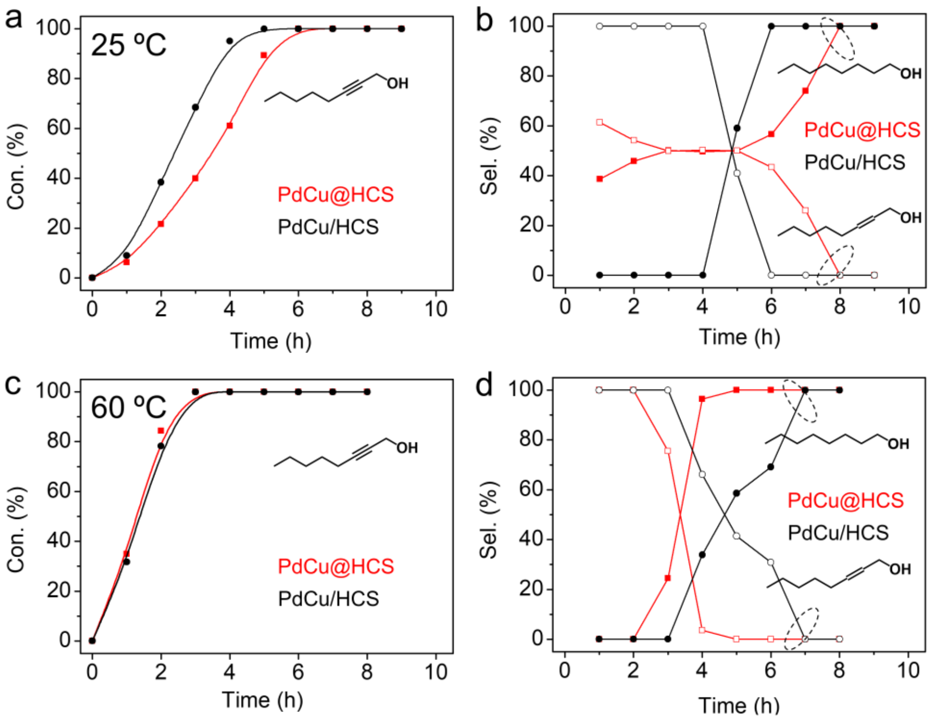

Figure S14. Hydrogenation of 2-Octyn-1-ol over PdCu@HCS and PdCu/HCS at (a,b) 25 ºC and (c,d) 60 ºC. Reaction conditions: H_2 balloon, 30 mg of catalyst, 1 mmol of substrate, 0.5 mmol of dodecane as internal standard, 5 mL of ethanol as solvent.

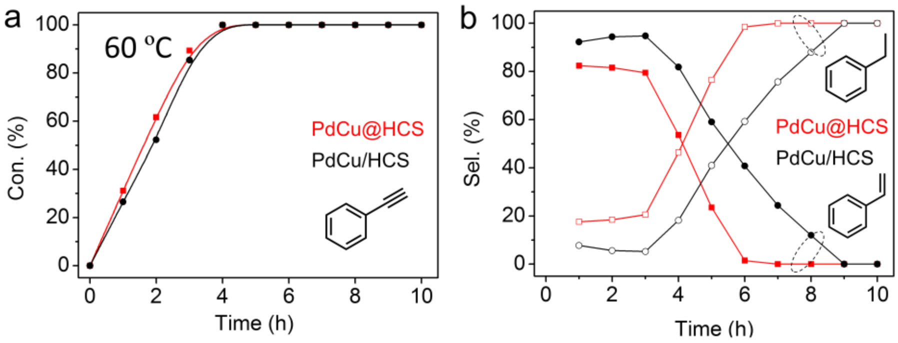

Figure S15. Conversion (a) and selectivity (b) for hydrogenation of phenylacetylene over PdCu@HCS and PdCu/HCS. Reaction conditions: H_2 balloon, 30 mg of catalyst, 1 mmol of substrate, 0.5 mmol of dodecane as internal standard, 5 mL of ethanol as solvent, 60 ºC.

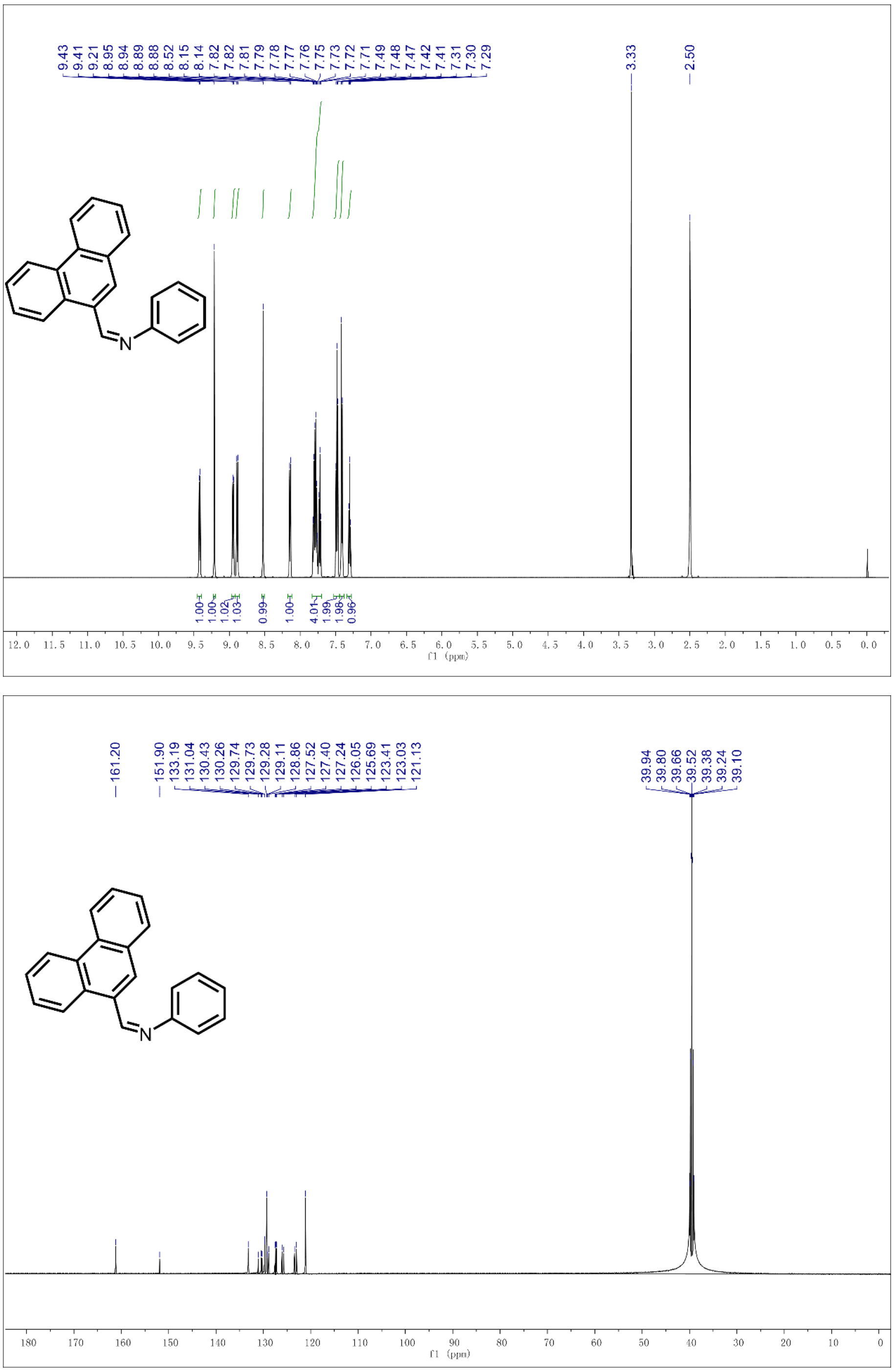

Figure 16. ^{1}H and ^{13}C NMR spectra of N-(9-Phenanthrenylmethylene)benzenamine.

***N*-(9-Phenanthrenylmethylene)benzenamine**: ^{1}H NMR (600 MHz, DMSO-d6, 298 K) δ (ppm) 9.42 (d, *J* = 7.6 Hz, 1H), 9.21 (s, 1H), 8.94 (d, *J* = 7.6 Hz, 1H), 8.89 (d, *J* = 8.3 Hz, 1H), 8.52 (s, 1H), 8.14 (d, *J* = 7.7 Hz, 1H), 7.86 – 7.75 (m, 3H), 7.72 (t, *J* = 7.3 Hz, 1H), 7.48 (t, *J* = 7.7 Hz, 2H), 7.41 (d, *J* = 7.3 Hz, 2H), 7.30 (t, *J* = 7.3 Hz, 1H). ^{13}C NMR (150 MHz, DMSO-d6, 298 K) δ (ppm) 161.20, 151.09, 133.19, 131.04, 130.43, 130.26, 129.74, 129.73, 129.28, 129.11, 128.88, 127.52, 127.40, 127.24, 126.05, 125.69, 123.41, 123.03, 121.13. HRMS (ESI^{+}) m/z calcd for $C_{21}H_{15}N + H^{+}$ 282.6316, found 282.1280.

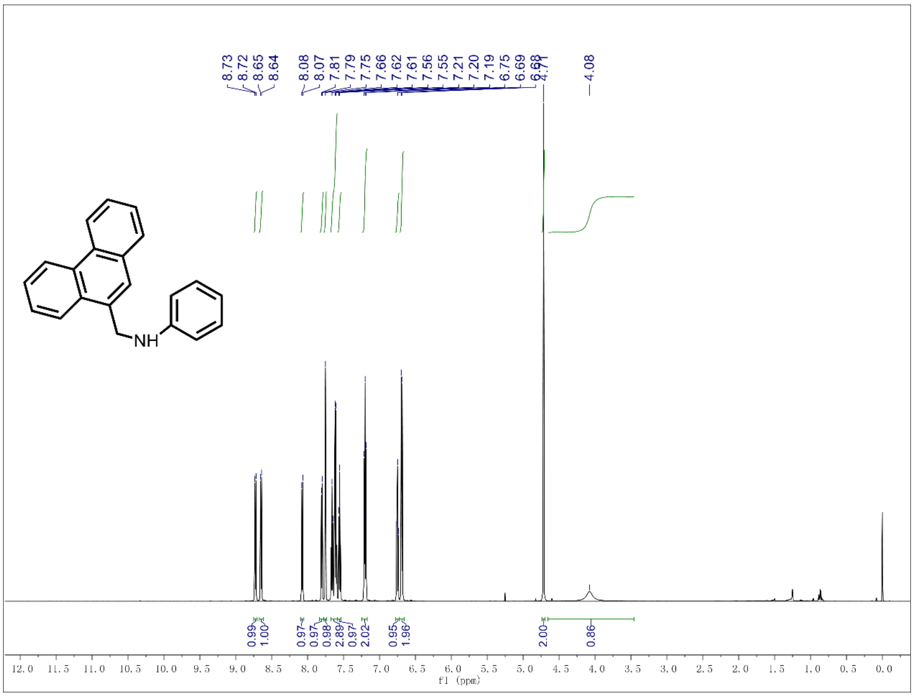

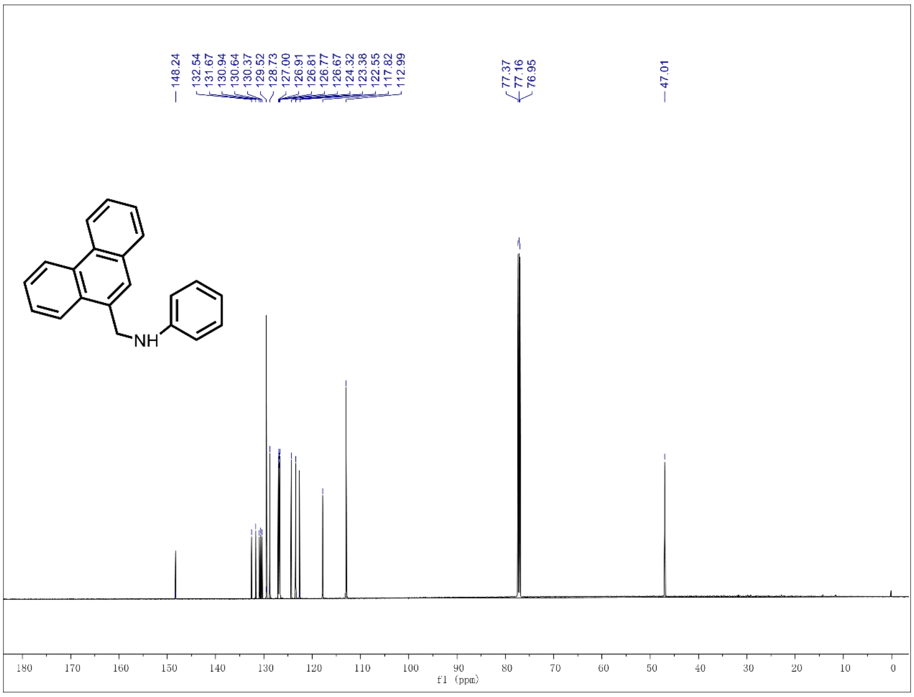

Figure 17. ^{1}H and ^{13}C NMR spectra of N-phenyl-9-Phenanthrenemethanamine.

***N*-phenyl-9-Phenanthrenemethanamine**: ^{1}H NMR (600 MHz, $CDCl_3$, 298 K) δ (ppm) 8.73 (d, J = 8.2 Hz, 1H), 8.65 (d, J = 8.3 Hz, 1H), 8.07 (d, J = 7.9 Hz, 1H), 7.80 (d, J = 7.6 Hz, 1H), 7.75 (s, 1H), 7.65 (d, J = 7.0 Hz, 1H), 7.61 (d, J = 7.0 Hz, 2H), 7.56 (d, J = 7.0 Hz, 1H), 7.25 – 7.14 (m, 2H), 6.75 (t, J = 7.3 Hz, 1H), 6.69 (d, J = 7.7 Hz, 2H), 4.71 (s, 2H), 4.08 (s, 1H). ^{13}C NMR (150 MHz, $CDCl_3$, 298 K) δ (ppm) 148.24, 132.54, 131.67, 130.94, 130.64, 130.37, 129.52, 128.73, 127.00, 126.91, 126.81, 126.77, 126.67, 124.32, 123.38, 122.55, 117.82, 112.99, 47.01. HRMS (ESI^{+}) m/z calcd for $C_{21}H_{17}N + H^{+}$ 284.1434, found 284.1436.

Table S2 The size of the molecules used in this work (according to the structure from Chem3D).

Molecule	Structure		Size (nm)
Styrene	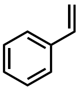	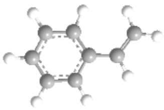	0.42 × 0.72
2-Vinylnapthalene	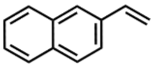	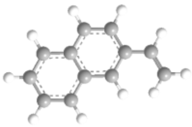	0.54 × 0.92
9-Vinylanthracene	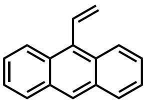	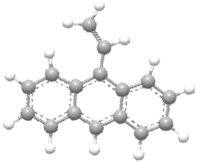	0.73 × 0.91
Phenylacetylene	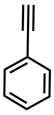	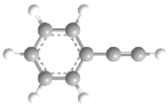	0.42 × 0.74
1-Ethynylnaphthalene	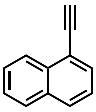	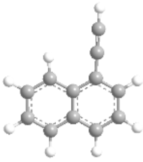	0.66 × 0.74
9-Ethynylphenanthrene	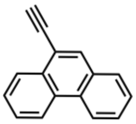	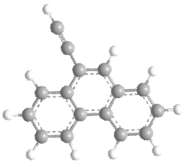	0.78 × 0.90
Nitrobenzene	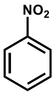	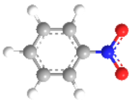	0.43 × 0.59
Phenanthrene-9-carboxaldehyde	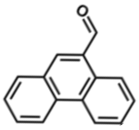	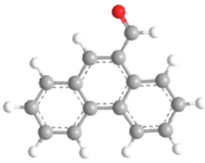	0.68 × 0.90
Imine	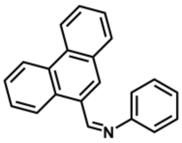	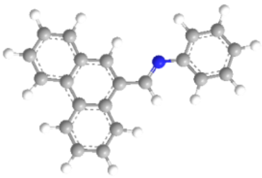	0.90 × 1.17

References

[1] a) K. Jiang, P. Wang, S. Guo, X. Zhang, X. Shen, G. Lu, D. Su, X. Huang, *Angew. Chem. Int. Ed.* **2016**, *55*, 9030-9035; b) K. H. Park, Y. W. Lee, S. W. Kang, S. W. Han, *Chem. Asian J.* **2011**, *6*, 1515-1519.
[2] R. Roa, W. K. Kim, M. Kanduc, J. Dzubiella, S. Angioletti-Uberti, *ACS Catal.* **2017**, *7*, 5604-5611.
[3] S. Angioletti-Uberti, Y. Lu, M. Ballauff, J. Dzubiella, *J. Phys. Chem. C* **2015**, *119*, 15723-15730.
